# How to Model Tendon-Driven Continuum Robots and Benchmark Modelling Performance

**DOI:** 10.3389/frobt.2020.630245

**Published:** 2021-02-02

**Authors:** Priyanka Rao, Quentin Peyron, Sven Lilge, Jessica Burgner-Kahrs

**Affiliations:** Continuum Robotics Laboratory, Department of Mathematical and Computational Sciences, University of Toronto Mississauga, Mississauga, ON, Canada

**Keywords:** soft manipulator, soft arm, tendon actuation, modelling, soft robot

## Abstract

Tendon actuation is one of the most prominent actuation principles for continuum robots. To date, a wide variety of modelling approaches has been derived to describe the deformations of tendon-driven continuum robots. Motivated by the need for a comprehensive overview of existing methodologies, this work summarizes and outlines state-of-the-art modelling approaches. In particular, the most relevant models are classified based on backbone representations and kinematic as well as static assumptions. Numerical case studies are conducted to compare the performance of representative modelling approaches from the current state-of-the-art, considering varying robot parameters and scenarios. The approaches show different performances in terms of accuracy and computation time. Guidelines for the selection of the most suitable approach for given designs of tendon-driven continuum robots and applications are deduced from these results.

## 1 Introduction

Continuum robots are slender and soft manipulators that are mainly characterized by their compliance and high dexterity. These properties together with their ability to follow non-linear trajectories and easy miniaturization enables applications in areas such as minimal-invasive surgery (Burgner-Kahrs et al., 2015) or inspection as well as assembly in confined spaces. Due to their inherent compliance, the deformation of continuum robots is highly affected by the presence of external forces. Such forces generally occur at the robot’s tip when interacting with the environment during deployment using different tools fixed at the end-effector. While in theory they offer an infinite number of degrees of freedom, only a subset of those can be actuated in practice. There exist several different actuation principles for continuum robots including both extrinsic and intrinsic methods. An overview of different actuation strategies for continuum robots is reviewed in Burgner-Kahrs et al. (2015).

**FIGURE 1:**
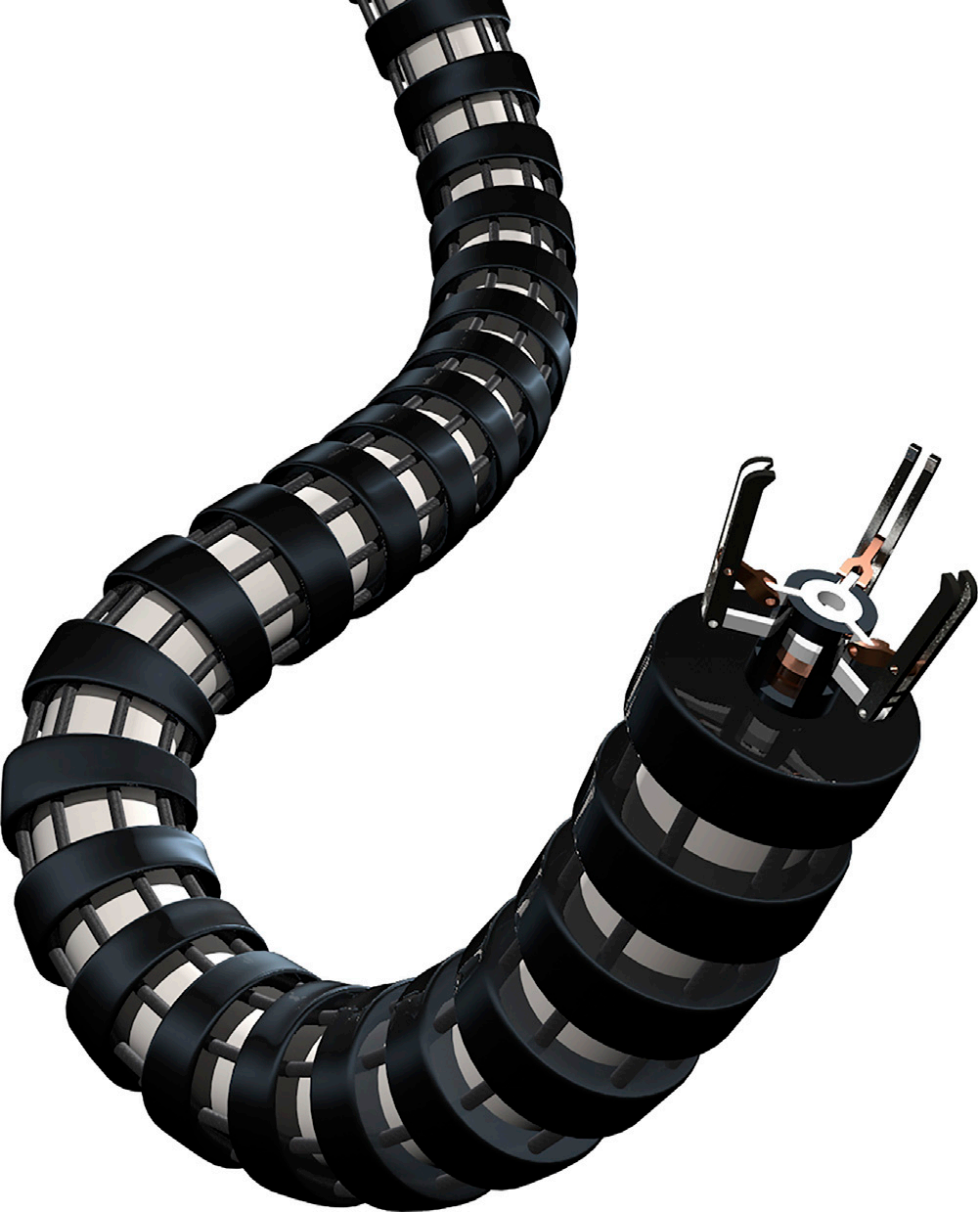
Example rendering of a tendon-driven continuum robot.

One commonly employed actuation principle is based on the use of tendons and these robots are called as tendon-driven continuum robots (TDCR, see Figure 1). Several tendons are routed along the robot’s flexible backbone. Actuation of the continuum robot is realized by pulling the tendons which results in bending motions. Tendon actuation is an extrinsic actuation principle, as no actuators are located inside the robot’s structure. The paper by (Hemami, 1985) is one of the first publications concerned with TDCR. This work proposes a flexible lightweight robot arm, which is composed of multiple flexible segments. The bending of each segment can be controlled using three tendons. As a result, the robot can adopt highly non-linear shapes which allows manipulation in confined spaces. A similar design was introduced by Immega and Antonelli (1995), who proposed a robotic manipulator to mimic the characteristic motions of an octopuses’ tentacle, profiting from their inherent compliance and dexterity. In comparison to the robot introduced by Hemami (1985), this manipulator can also extend and retract. The first tendon-driven mechanisms in the context of medical applications are introduced by Webster (1988) and Peirs et al. (2003).

**FIGURE 2:**
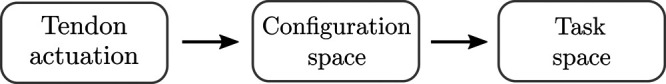
The modelling framework of a TDCR that considers the tendon actuation to calculate the resulting configuration space parameters. The configuration space parameters can then be used to obtain the corresponding backbone shape in 3D space.

Over the recent years, many more similar TDCR designs were introduced. In order to design, analyze and control such robotic manipulators, analytic models are required. They allow to calculate the resulting deformed shape of the robot given specific actuation inputs. The actuator space, that describes the actuation of the robot’s tendons, is mapped to a configuration space, which parameterizes the robot’s shape. The configuration space parameters are then mapped to the resulting position and orientation of the shape in task space.

While the configuration space representation theoretically requires an infinite number of parameters due to the continuous nature of their backbones, a reduced set of parameters can be chosen to represent the robot’s backbone. Furthermore, different assumptions of increasing complexity regarding the kinematics and statics of the robot’s backbone and tendons can be included in the mapping between actuation and configuration space. As a result, existing kinematic and static models are often tailored to a specific robot design and make trade-offs between accuracy and computation speed depending on the envisioned application. For instance, continuum robot design requires models which describe the robot behaviour accurately, without specific requirements on the computation time. Robot control is less demanding in terms of accuracy, since model errors can be compensated by sensor feedback, but requires fast computation time.

Although there are various dynamic models to address these challenges (Rucker and Webster, 2011a; Rone and Ben-Tzvi, 2013), we focus the scope of this paper to kineto-static models as these robots are generally operated operated in a quasi-static fashion (Burgner-Kahrs et al., 2015).

Thus, the major challenge when modelling TDCR is to select a viable kinematic and static modelling, choosing suitable kinematic and static assumptions in combination with a corresponding configuration space parameterization with respect to desired accuracy and computation time. We aim to address these challenges throughout this work.

While data-driven models (e.g. Giorelli et al. (2015); Xu et al. (2017)) have been proposed to bypass these challenges, they cannot be applied feasibly for design related tasks. Therefore, we focus the scope of our paper to cover analytical methods.

The contributions of this paper are twofold. First, an exhaustive review of existing kinematic and static analytic models for TDCR is presented. While there already exist a handful of modelling reviews for general continuum robots (Webster and Jones, 2010; Walker, 2013; Chirikjian, 2015; Walker et al., 2016), no review focuses on tendon-driven continuum robots and all their different possible modelling approaches yet. We organize and classify TDCR models based on their representation of the continuum robot’s flexible backbone as well as their kinematic and static assumptions. Second, guidelines for choosing a sufficient and feasible model w.r.t. different scenarios are generated through a numerical case study. In order to do so, the most relevant state-of-the-art kinematic models are implemented in both C++ and MATLAB, partially leveraging and extending their formulations to account for general robot structures and configurations. The code is made openly available to ease the process of developing such models. Based on the implementation, case studies are conducted to compare and benchmark the different model performances for different design parameters and assumptions with respect to their accuracy and computation times. Eventually, by introducing a common terminology and providing open source implementations of representative models, this work provides resources to evaluate any newly proposed modelling approach w.r.t. existing approaches in a straightforward manner.

## 2 Mechanical Structure of TDCR

As mentioned above, TDCR utilize tendons routed along their backbone that are fixed at predefined locations along the robot’s arc length. They can consist of multiple stacked **segments**, where each segment end is defined by the termination of one or multiple tendons. When pulling these tendons, a load will be applied to the compliant backbone and the corresponding segment will bend in the direction of the routed tendon.

**FIGURE 3:**
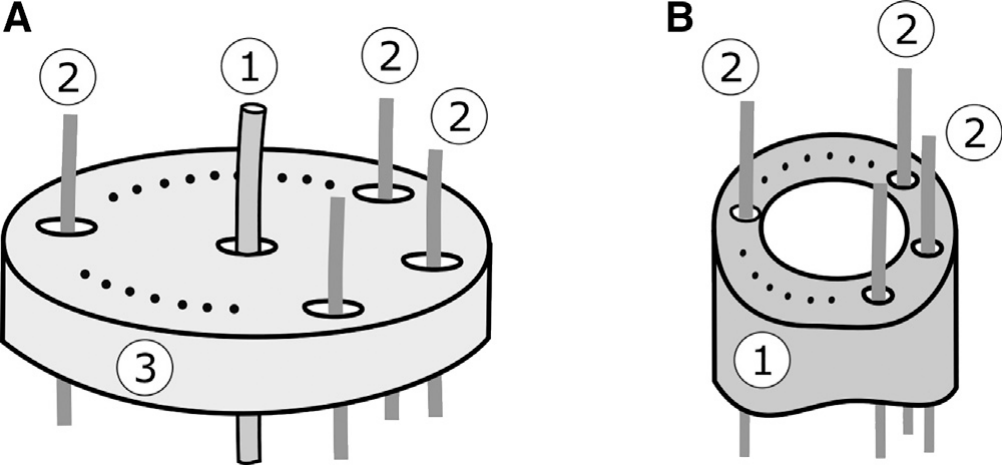
Schematics of the two typical TDCR design structures: **(A)** A primary flexible, slender backbone (1) is employed, while equally distributed spacer disks (3) are used to route tendons along the backbone (2); **(B)** A larger diameter flexible backbone (1) is used that already features inner lumens to guide the tendons (2) without the need of additional spacer disks.

Analyzing the current state-of-the-art of TDCR manipulators, two distinct design structures can be identified as shown in Figure 3. The first structure (Figure 3A) consists of a primary flexible, slender backbone. A finite number of spacer disks is equally distributed along the whole length of the backbone. Guiding holes within the spacer disks are used to route the tendons along the robot backbone. For example, this structure is used in papers by (Rucker and Webster, 2011a) and (Simaan et al., 2004). This design realizes a **partially constrained tendon path**, in which the tendon path is straight within a **subsegment** (i.e. between two individual spacer disks), forming a series of line segments along the backbone.

The second design, shown in Figure 3B, uses a backbone which usually has a larger diameter and already features inner lumens to guide the tendons without the need of separate spacer disks (Camarillo et al., 2008b; Kato et al., 2015; Xu et al., 2015). In contrast to the first design, here a **fully constrained tendon path** is realized. It is assumed that the tendons follow a continuous curve, parallel to the backbone.

For both designs, several components have been considered for realizing the flexible backbone: Either as a single piece flexible tube or rod that has a uniform stiffness (Rucker and Webster, 2011a), multiple concentrically nested flexible tubes that lead to multiple segments each with a different uniform stiffness (Amanov et al., 2019), a flexible tube or rod with a cutting pattern to allow for non uniform stiffness (Camarillo et al., 2008b), or a discrete assembly of multiple stacked compliant joints (Kato et al., 2015). While Amanov et al. (2019) realizes a TDCR structure with segments of variable lengths, we will focus on segments with fixed lengths throughout this work. On top of that, different materials have been investigated for the robot’s backbone, ranging from steel to Nitinol and polymer, leading to vastly different stiffness properties. While special care can be taken when choosing the material of the tendons and the guiding channels to minimize friction and possible stretching of the tendons. However, these effects are often neglected, as their impact on the modelling accuracy is usually considered to be orders of magnitudes lower as the actuation. Therefore, throughout this work, both friction between tendons and the robot’s backbone or spacer disks as well as tendon elongation are not considered.

While in theory, the tendons can be employed using many different routing paths along the backbone, e.g. helical (Starke et al., 2017) or converging (Oliver-Butler et al., 2019) paths, we focus on generalising models for straight tendon routing throughout this work as the most common routing method employed. For each segment, any number of tendons can be employed and routed along the robot’s backbone, while at least two are needed to allow for bending in one degree of freedom (planar bending) and three for bending in two degrees of freedom (spatial bending), respectively.

**FIGURE 4:**
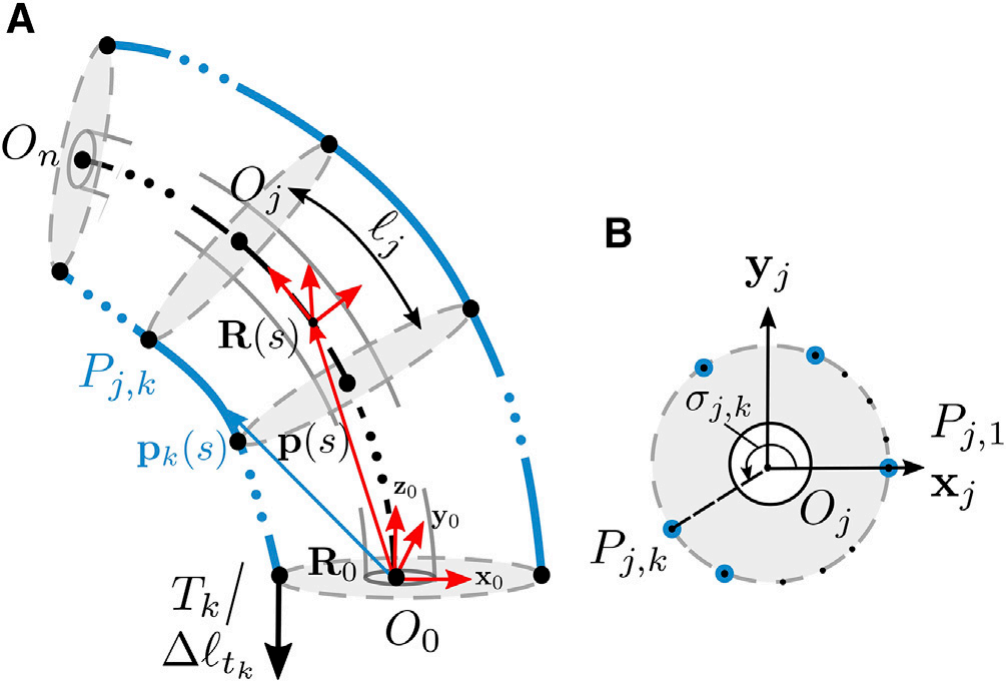
**(A)** Diagrammatic representation of one segment of a TDCR, actuated by tension $T_{k}$ or tendon displacements $\text{Δ}\ell_{t_{k}}$ on the *k*th tendon. Dashed line indicates the backbone centreline while the blue solid lines denote the tendons **(B)** The *k*th tendon passes through the *j*th cross section at $P_{j,k}$. The tendons are labelled in an anticlockwise manner, arranged radially around the center of the cross section, $O_{j}$.

In order to mathematically describe the TDCR structure, we introduce a common nomenclature that is used throughout this work and can be applied to both designs discussed in this section. Figure 4A shows a typical TDCR considered in this review, with equidistant cross-sections. We note, that while the proposed nomenclature is described using one single bending segment, it can be extended to multi-segment robots in a straight-forward manner as described in the later sections. Tendons are routed along the backbone, at a distance $r_{d}$ from the center. The point of their termination marks the end of the segment of length ℓ. The segment is further divided into a series of *n* subsegments of length $\ell_{j}$, where j=1,2...n with $O_{j-1}$ indicating the base of each subsegment. The *j*th subsegment refers to the part of the robot (backbone and tendons) enclosed between $O_{j-1}$ and $O_{j}$.

Each point on the backbone is parameterized along its arc length s∈[0,ℓ] by a locally defined frame. The local frame is constructed such that the local z axis is tangent to the backbone and the local x axis joins the center of the backbone to the first tendon, numbered 1. The backbone position is described by the position vector $p\left(s\right)\in {\mathbb{R}}^{3}$ indicating the origin of the local frame w.r.t. to the base frame. The orientation of the local frame w.r.t. to the base frame is represented by the rotation matrix R(s)∈SO(3). Overall, the pose of the local frame can be represented by a homogeneous transformation matrix, T(s)∈SE(3).

$$T\left(s\right)=\left[\begin{array}{cc} R\left(s\right) & p\left(s\right)\\ 0 & 1 \end{array}\right]$$ (1)

The rotation frame attached at the base of subsegment *j* at $O_{j-1}$ is also denoted by $R_{j-1}\in \text{SO}\left(3\right)$ formed by the local axes $x_{j-1}$, $y_{j-1}$ and $z_{j-1}$. The *m* tendons are numbered in an anti-clockwise manner, as shown in Figure 4B. The position vector $p_{k}\left(s\right)\in {\mathbb{R}}^{3}$ denotes the location of tendon *k* corresponding to a particular value of *s*. The point of intersection of this tendon with the cross section passing through $O_{j}$ is denoted $P_{j,k}$. Considering the tendon at the constraining locations, which depend on the chosen design (partially or fully constrained tendon path), all the tendons are considered to be equidistant from the backbone, and are arranged uniformly along the backbone circumference. The position of the tendons at any cross section can then be expressed in polar coordinates with respect to the local X axis as $\left(r_{k},\sigma_{k}\right)$, where $r_{k}=r_{d}\forall k$ and $\sigma_{k}=\left(k-1\right)2\pi /m$.

## 3 Continuum Backbone Parameterization

**FIGURE 5:**
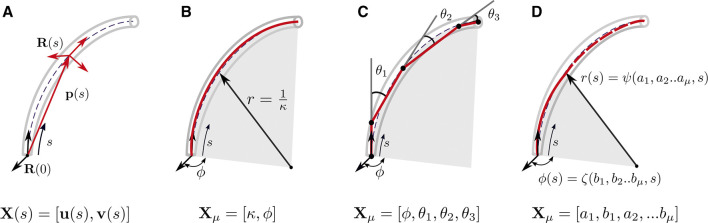
Diagrammatic representation of various kinematic frameworks used to describe the backbone. The backbone parameters required are denoted by X(s) for distributed backbone parameterization and $X_{\mu }$ for lumped backbone parameterization. **(A)** Variable curvature representation use to describe the position p(s) using an attached frame, whose orientation is represented by R(s) **(B)** Arc parameters (κ,ϕ) used to describe a segment with constant curvature without torsion **(C)** Pseudo-rigid body 3 R model approximating the backbone as a four link serial manipulator **(D)** Representing the backbone parameters using shape functions $\psi \left(a_{1},a_{2},..s\right)$ and $\eta \left(b_{1},b_{2}..s\right)$ in the modal approach.

Several approaches have been proposed to represent the shape of continuum robots. These methods lead to different expressions of the backbone curvature, and therefore the resulting position and orientation. We classify the existing representations as distributed and lumped backbone parameterizations by adapting the categorization used in Rone and Ben-Tzvi (2014a). In distributed parameterization, the curve is represented as a continuous function of *s*. It’s state-space has been represented by X(s). In lumped parameterization, the backbone pose is represented by a finite set of parameters and its state space by $X_{\mu }$. Figure 5 summarizes the major backbone parameterizations used in literature.

### 3.1 Distributed Backbone Parameterization

As the flexible backbone follows a continuous curve, an infinite number of parameters are theoretically required to describe it in task space. Doing so with state-space vectors that are a continuous function of the distance along the backbone is referred to as distributed parameter modelling. As it makes no assumptions on the backbone shape, it is a geometrically exact representation and can account for any arbitrary variations in curvature by differential equations. Therefore, it is referred to as the *variable curvature representation*.

The strain variables $u\left(s\right)\in {\mathbb{R}}^{3}$ and $v\left(s\right)\in {\mathbb{R}}^{3}$ are used to represent the rate of change of the rotation and position matrix respectively. The former, u(s) denotes an infinitesimal rotation of the attached frame R(s), while v(s) denotes the linear velocity of the rigid body expressed in rigid body coordinates. They can be used to represent the backbone parameters, X(s) as shown in Figure 5A.

$$\frac{\text{d}R\left(s\right)}{\text{d}s}=R\left(s\right){\left[u\left(s\right)\right]}_{\times }$$ (2)

$$\frac{\text{d}p\left(s\right)}{\text{d}s}=R\left(s\right)v\left(s\right)$$ (3)

When the backbone is assumed to be inextensible and shearless, there is no axial deformation and therefore, $v\left(s\right)={\left[0,0,1\right]}^{T}$. The operator ${\left[.\right]}_{\times }$ represents the mapping from ${\mathbb{R}}^{3}$ to so (3) as a skew symmetric matrix, ${\left[u\left(s\right)\right]}_{\times }$ given by

$${\left[u\left(s\right)\right]}_{\times }=\left[\begin{array}{ccc} 0 & -u_{z} & u_{y}\\ u_{z} & 0 & -u_{x}\\ -u_{y} & u_{x} & 0 \end{array}\right].$$ (4)

The complete robot shape and orientation in 3D are obtained by integrating Eq. (2) and (3). It is not possible to perform the integration analytically for the entire backbone due to its nonlinear deformations. Therefore, it requires numerical integration (Jones et al., 2009; Dehghani and Moosavian, 2011; Dehghani and Moosavian, 2013a). For only the tip orientation matrix, a closed analytical solution has been derived by Hsiao and Mochiyama (2017). Computing the rotation matrix by integrating Eq. (2) does not ensure that the properties of so(3) are preserved. However, the numerical errors have been shown to be small for the small curvatures experienced by these robots (Rucker and Webster, 2011b). Moreover alternate representations of the orientation using quaternions (Rucker, 2018; Till et al., 2019) can be used to conserve the properties of the special orthogonal group.

### 3.2 Lumped Backbone Parameterization

In lumped parameter modelling, the backbone is discretized and represented with a finite set of parameters. The required infinite backbone parameters are reduced by making assumptions on the backbone shape using geometry. These assumptions lead to a trade-off between the complexity and accuracy because while they simplify the backbone description, there is an accompanying loss of information as interpolation is required to obtain each point on the backbone. By contrast, the distributed parameterization describes each point on the backbone without interpolation and does not lead to any such loss.

We refer to the finite set of backbone parameters by $X_{\mu }$. In the literature, these assumptions have been made by either parameterizing the curve properties or approximating the backbone as a series of rigid links connected by joints with torsional springs. In addition to methods applied to TDCR designs, the referred works include those that consider a continuous backbone and actuator lengths as parameters as it is directly applicable to TDCR modelling.

#### 3.2.1 Constant Curvature (CC) Assumption

This assumption forms the basis for a common approach to parameterizing the backbone. The backbone is assumed to be a series of mutually tangent sections, which are approximated as arcs with constant curvatures. This approach has also been referred to as the piecewise constant-curvature (PCC) approximation. The constant curvature assumption can be applied to either the entire segment or each subsegment. To distinguish between the two, we refer to them as CC and CC_sub_ respectively. The term ’section’ has been used here to refer to the segment or subsegment, approximated as an arc.

If the effects of torsion are neglected, the section can be assumed to undergo planar deformations. As depicted in Figure 5B, its backbone parameters can be represented by $X_{\mu }=\left[\kappa ,\phi \right]$, where κ is the curvature and ϕ the angle its bending plane makes with a reference axis. The task space coordinates can be obtained from the arc parameters by various formulations like arc geometry, Denavit–Hartenburg (D-H) parameters, Frenet-Serret frames, or exponential coordinates. These representations have been derived and compared in Webster and Jones (2010), who show that they are equivalent as they yield the same mapping between configuration and task space. The approach using arc geometry to obtain the resulting homogeneous transformation matrix is detailed below. The position of any point on the arc, with respect to the base of the arc at a distance *s* is given by

$$p\left(s\right)=\left[\begin{array}{c} \frac{\text{cos}\phi }{\kappa }\left(1-\text{cos}\left(\kappa s\right)\right)\\ \frac{\text{sin}\phi }{\kappa }\left(1-\text{cos}\left(\kappa s\right)\right)\\ \frac{1}{\kappa }\text{sin}\left(\kappa s\right) \end{array}\right].$$ (5)

The above involves a rotation about the **y** axis by (κs), which is also denoted by θ(s) (Simaan et al., 2004; Simaan et al., 2009; Goldman et al., 2014). An alternative, proposed by Hannan and Walker (2003) to the curve representation is to use the components of the curvature along the **x** axis ($\kappa_{x}$) and **y** axis ($\kappa_{y}$) of the local frame (Camarillo et al., 2008a; Dehghani and Moosavian, 2013b; Rone and Ben-Tzvi, 2014b). The curvature and bending angle is then represented as

$$\kappa =\sqrt{\kappa_{x}^{2}+\kappa_{y}^{2}},$$ (6)

$$\phi =\text{atan}2\left(\kappa_{y},\kappa_{x}\right).$$ (7)

Equating ϕ to zero gives the resulting coordinates in the **xz** plane of the frame attached to the bast of the section. Depending on whether a segment (Hannan and Walker, 2003; Xu et al., 2015; Ouyang et al., 2016; Dalvand et al., 2018; Mishra et al., 2018) or a subsegment (Kutzer et al., 2011; Mahl et al., 2013; Kato et al., 2015; Kato et al., 2016) is being approximated as a circular arc, the backbone parameters are represented by [κ,ϕ] or $\left[\kappa_{j},\phi_{j}\right]$ respectively. Therefore, CC and CC_sub_ require 2 and 2n parameters respectively. The coordinate frame of any point on the arc, expressed with respect to the frame attached to the base of the section can be obtained by two consecutive rotations about the **z** and **y** axis. The resulting transformation matrix is given by

$$T\left(s\right)=\left[\begin{array}{cc} R_{z}\left(\phi \right)R_{y}\left(\kappa s\right)R_{z}\left(-\phi \right) & p\left(s\right)\\ 0 & 1 \end{array}\right]$$ (8)

where

$$R_{z}\left(\phi \right)=\left[\begin{array}{ccc} \text{cos}\left(\phi \right) & -\text{sin}\left(\phi \right) & 0\\ \text{sin}\left(\phi \right) & \text{cos}\left(\phi \right) & 0\\ 0 & 0 & 1 \end{array}\right]$$ (9)

$$R_{y}\left(\kappa s\right)=\left[\begin{array}{ccc} \text{cos}\left(\kappa s\right) & 0 & \text{sin}\left(\kappa s\right)\\ 0 & 1 & 0\\ -\text{sin}\left(\kappa s\right) & 0 & \text{cos}\left(\kappa s\right) \end{array}\right].$$ (10)

In case the backbone is subject to torsion, an additional parameter ϵ is introduced to account for the twist (Rone and Ben-Tzvi, 2014a; Yuan et al., 2019) in the arc in CC_sub_. The position vector of the section remains the same as given in equation Eq. (5). However, the rotation matrix includes an additional rotation about the current **z** axis by (ϵ−ϕ). Li and Rahn (2002) study the geometrical limits that allowed the validity of the constant curvature assumption for a single subsegment in the planar case. Camarillo et al. (2008b) prove theoretically that the backbone is a circular arc under certain planar loading conditions.

#### 3.2.2 Pseudo-Rigid Body Representation (PRB)

In the PRB approach, the backbone along a subsegment is modelled as a series of rigid links connected by torsion springs. Roesthuis and Misra (2016) approximate the backbone as a series of rigid links connected by a single torsional spring. However, the optimal stiffness of the springs and length of each link are dependent on the values of force and moment experienced by the robot (Su, 2009). As continuum robots can experience large deformations due to applied load, the 3R model, proposed by (Su, 2009) has been used to model TDCRs as its parameters are load independent. It is applied to the planar case by Huang et al. (2018) and Khoshnam et al. (2015), and further extended to the 3D case by Huang et al. (2019). The subsegment is approximated by four rigid links, as shown in Figure 5D, with the first and last link tangent to the local **z** axis.

The length of each link i=0,1,2,3 is defined with respect to the subsegment length using a factor $\gamma_{i}$. Each subsegment, is assumed to bend in a plane rotated about an angle $\phi_{j}$ about the **z** axis. The transformation matrix from the end of a link *i* to its base in the plane of bending is given by

$$T_{j,i}=\left[\begin{array}{cccc} \text{cos}\left(\theta_{j,i}\right) & 0 & \text{sin}\left(\theta_{j,i}\right) & \gamma_{j,i}\ell_{j}\text{sin}\left(\theta_{j,i}\right)\\ 0 & 1 & 0 & 0\\ -\text{sin}\left(\theta_{j,i}\right) & 0 & \text{cos}\left(\theta_{j,i}\right) & \gamma_{j,i}\ell_{j}\text{cos}\left(\theta_{j,i}\right)\\ 0 & 0 & 0 & 1 \end{array}\right].$$ (11)

The value of $\theta_{j,0}$ is 0 as it remains parallel to the $z_{j-1}$ axis. Using the above, the transformation matrix from the base of the next subsegment to the current can be given by

$$T_{j}^{j+1}=T_{z}\left(\phi_{j}\right)T_{j,0}T_{j,1}T_{j,2}T_{j,3}T_{z}\left(-\phi_{j}\right)$$ (12)

where $T_{z}\left(\phi \right)$ represents a rotation about the **z** axis by ϕ. The rotation by (−ϕ) ensures that there is no twist in the model. Therefore, the above model, proposed by Huang et al. (2019) can only account for in-plane bending of a subsegment, and cannot account for external forces. In order to account for this external force, we propose the inclusion of an additional parameter, $\epsilon_{j}$ to account for the twist in each subsegment. Eq. (12) is then post-multiplied by a rotation about the **z** axis by $\epsilon_{j}$.

Ashwin and Ghosal (2019) and (2021) propose a backbone representation specific to TDCRs, where the backbone, along with the tendons, is modelled as a series of two adjoined perpendicular four-bar links. In a given subsegment, the backbone forms the crank with length $\ell_{j}$ while the corresponding portion of the enclosed tendon with length $\ell_{t_{k,j}}$ behaves like the second crank. The portion of the disk connecting the bottom of the backbone section to the tendon is the fixed link with length equal to the disk radius $r_{d}$, while the upper connection behaves as a coupler with the same length.

#### 3.2.3 Modal Approach

In the modal approach, the backbone is expressed in terms of curve parameters, which are linear combinations of modal shape functions (MSFs). The MSFs are continuous functions, dependent on *s* and are used to describe a given curve parameter c(s).

$$c\left(s\right)=\overset{n}{\underset{r=1}{\sum }}a_{r}{\text{Φ}}_{r}\left(s\right)$$ (13)

where $a_{r}$ is the *r*th coefficient and ${\text{Φ}}_{r}$ is the *r*th MSF. The number of curve parameters required depends on the backbone parameters required to describe the system. For in-plane bending, two curve parameters (eg. curvature κ(s) and bending plane angle ϕ(s)) are used to describe the curve, shown in Figure 5C. For planar bending (ϕ(s)=0), one backbone parameter is sufficient to describe the curve. In (Gravagne and Walker, 2000a; Gravagne and Walker, 2000b; Gravagne and Walker, 2002), the bending angle θ(s) is expressed as either a function of natural or wavelet basis functions. The former consists of box functions that are non-zero within a segment, resulting in the segments being represented independently. The wavelet basis functions seek to correct this through the use of the Haar wavelet family as shape functions.

Godage et al. (2011a) express the backbone coordinates as polynomial functions of the actuator lengths. Each MSF is derived using the Taylor series expansion of the curve parameters, expressed in terms of the input actuator lengths (Godage et al., 2011b; Godage et al., 2015). Polynomial approximation of curve parameters provides an alternative option for the shape functions. Santina and Rus (2020) approximate the curvature as a polynomial in terms of the parameter *s*.

## 4 Kinematic Modelling

Kinematic modelling involves mapping the tendon lengths to the corresponding backbone position and orientation. The tendon lengths ($\ell_{t_{1}},\ell_{t_{2}}\ldots \ell_{t_{m}}$) enclosed by a segment or the change in tendon lengths ($\text{Δ}\ell_{t_{1}},\text{Δ}\ell_{t_{2}}\ldots \text{Δ}\ell_{t_{m}}$) are considered as inputs from the actuators. Closed-form forward kinematics have been developed for the constant curvature assumption to obtain the pose of the entire backbone that are described here.

### 4.1 Fully Constrained Tendon Path

When the constant curvature assumption is used, the arc parameters (κ,ℓ,ϕ) are mapped to the input actuator lengths represented by $q=\left[\ell_{t_{1}}\ell_{t_{2}}..\ell_{t_{m}}\right]$. The tendons run parallel to the central arc as secondary arcs, whose radius is given by the relation

$$r_{k}=r-r_{d}\text{cos}\left(\sigma_{k}-\phi \right)$$ (14)

where $r_{k}$ is the radius of the arc formed by the tendon *k* and *r* is the radius of the backbone curve, given by (1/κ). The value of $\sigma_{k}$, as defined earlier is (k−1)2π/m. Since the angle θ enclosed by both the central and secondary arcs is same, multiplying Eq. (14) by θ gives the relation between the actuator lengths and central arc parameters as

$$\ell =\ell_{t_{k}}+\theta r_{d}\text{cos}\left(\sigma_{k}-\phi \right).$$ (15)

As the segment requires only two degrees of freedom, one of the tendons becomes redundant for three-tendon actuation, experiencing the following constraint

$$\ell =\frac{\ell_{t_{1}}+\ell_{t_{2}}+\ell_{t_{3}}}{3}.$$ (16)

Using the above relations, a closed-form solution for the three-tendon actuation is given by (Hannan and Walker, 2003; Webster and Jones, 2010)

$$\phi ={\text{tan}}^{-1}\left(\frac{3\left(\ell_{t_{3}}-\ell_{t_{2}}\right)}{\sqrt{3}\left(\ell_{t_{2}}+\ell_{t_{3}}-2\ell_{t_{1}}\right)}\right)$$ (17)

$$\kappa =\frac{2\sqrt{\ell_{t_{1}}^{2}+\ell_{t_{1}}^{2}+\ell_{t_{1}}^{2}-\ell_{t_{1}}\ell_{t_{2}}-\ell_{t_{1}}\ell_{t_{3}}-\ell_{t_{2}}\ell_{t_{3}}}}{r_{d}\left(\ell_{t_{1}}+\ell_{t_{2}}+\ell_{t_{3}}\right)}.$$ (18)

To avoid singularities arising in the position coordinates when κ=0, Godage et al. (2011a); Godage et al. (2011b) and Godage and Walker (2015) express the transformation matrix of the end-effector in terms of the tendon lengths. The relations obtained in Eq. (18) and (17) is substituted in Eq. (9). The trigonometric terms are approximated as polynomials in terms of the tendon lengths $\ell_{t}$ using the multivariate Taylor series expansion. The resulting transformation matrix requires 9 MSF (six from rotation and three from position) due to redundancies.

$$T\left(\ell_{t},s\right)=\left[\begin{array}{cc} R\left(\ell_{t},s\right) & p\left(\ell_{t},s\right)\\ 0 & 1 \end{array}\right]$$ (19)

Using the PRB model described in 3.2.2, Ashwin and Ghosal (2021) propose the forward kinematics of a TDCR. A subsegment is approximated as a four-bar mechanism. It is proven that the configuration of the backbone in static equilibrium is one where the change in coupler angle from the straight configuration is minimized. In 3D, a series of two four-bar mechanisms, perpendicular to each other are considered. Numerical optimization is used to calculate the configuration that results in the minimum change in the coupler angle from the straight configuration.

### 4.2 Partially Constrained Tendon Path

For the constant curvature assumption, the same approach as detailed for the fully constrained tendon path can be applied. The relation between *n* subsegments having partially constrained tendon length ${\hat{\ell }}_{t_{k}}$ and fully constrained tendon length $\ell_{t_{k}}$ in a segment is given by (Allen et al., 2020)

$${\hat{\ell }}_{t_{k}}=2\frac{\ell_{t_{k}}n}{\theta }\text{sin}\left(\frac{\theta }{2n}\right).$$ (20)

The tendon lengths calculated for a partially constrained design equals the length calculated for a fully constrained design when n→∞, where *n* is the number of subsegments, as proved in the paper by Gravagne and Walker (2000c). While the expression for ϕ remains the same as the fully constrained case, the length and curvature can be expressed as (Jones and Walker, 2006b; Webster and Jones, 2010)

$$\ell =\frac{nr_{d}\left({\hat{\ell }}_{t_{1}}+{\hat{\ell }}_{t_{2}}+{\hat{\ell }}_{t_{3}}\right)}{2C}{\text{sin}}^{-1}\left(\frac{C}{3nr_{d}}\right)$$ (21)

$$C=\sqrt{{\hat{\ell }}_{t_{1}}^{2}+{\hat{\ell }}_{t_{2}}^{2}+{\hat{\ell }}_{t_{3}}^{2}-{\hat{\ell }}_{t_{1}}{\hat{\ell }}_{t_{2}}-{\hat{\ell }}_{t_{1}}{\hat{\ell }}_{t_{3}}-{\hat{\ell }}_{t_{2}}{\hat{\ell }}_{t_{3}}}$$ (22)

$$\kappa =\frac{{\hat{\ell }}_{t_{2}}+{\hat{\ell }}_{t_{3}}-2{\hat{\ell }}_{t_{1}}}{\left({\hat{\ell }}_{t_{1}}+\hat{\ell_{t_{2}}}+{\hat{\ell }}_{t_{3}}\right)r_{d}\text{sin}\left(\phi \right)}.$$ (23)

An alternate representation is proposed by Allen et al. (2020) to avoid singularities in representing the straight configuration (when κ=0). The arc is expressed as a rotation of magnitude θ about ρ. The magnitude of ρ is equal to the radius of curvature of the arc and has two components, $\rho_{x}$ along the **x** axis and $\rho_{y}$ along the **y** axis. The angle between ρ and $\rho_{y}$ is given by ϕ. The curve is then, instead represented by $\left[\theta ,\rho_{y}\right]$. Similar to the approach by Godage et al. in representing the curve parameters by eliminating trigonometric functions, Barrientos-Diez et al., (2021) linearize the expression for tendon length, given in Eq. (20) by using the first order of the Taylor series expansion.

### 4.3 Extending to Multiple Segments

When there are *g* segments stacked on top of one other, the behaviour of each segment can be considered independently. Then, the transformation matrices of each segment can be multiplied consecutively to obtain the forward kinematics of the robot. However, there is coupling between the segments due to multiple sets of tendons passing through them. Jones and Walker (2006a) propose a tangle/untangle algorithm that takes into account the effect of this coupling between segments to predict the behaviour of the multisegment robot.

## 5 Static Modelling

Static modelling involves the mapping of tendon tension and external forces to the robot backbone shape. We consider the tensions $T_{k}$ applied on each tendon. The relationship between the tendon lengths and tensions can be found in Oliver-Butler et al. (2019) and Rone and Ben-Tzvi (2013). The tendon forces are dependent on whether they are assumed to be fully or partially constrained. In addition, we consider an external force $F_{ext}$ acting at the tip of the segment. The static model is obtained by writing the net force and moment equilibrium equations between the tendon and external forces to find the resulting internal forces and moments produced. Then, these forces are coupled with the backbone parameterization detailed in Section II using constitutive and equilibrium equations. These equations capture the elastic behaviour of the backbone and the resulting equations can be solved to obtain the pose of the backbone in task space.

### 5.1 Forces due to Constraints on the Tendon Path

Tendons apply two major types of forces on the backbone - termination forces at their point of attachment and distributed forces along the backbone. The magnitude and direction of these forces is determined the assumed tendon path.

#### 5.1.1 Forces due to Fully Constrained Tendon Path

**FIGURE 6:**
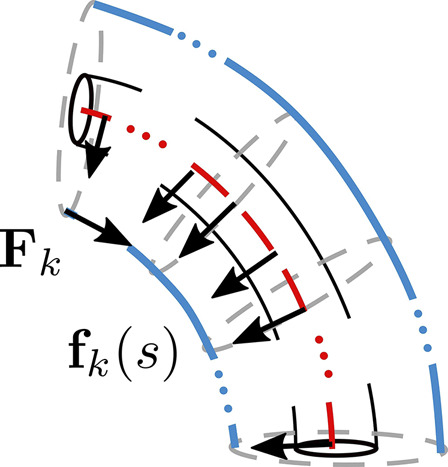
Representation of uniformly distributed force $f_{k}\left(s\right)$ and tendon termination force $F_{k}$ exerted on the backbone, by assuming that tendon *k* follows a continuous path.

The *k*th tendon applies a force $F_{k}$ at the point of termination, shown in Figure 6. It is equal and opposite to the internal force, $n_{k}\left(\ell \right)$ experienced by the tendon and given by (Rucker and Webster, 2011a)

$$F_{k}=-T_{k}\frac{p_{k}^{\prime }\left(\ell \right)}{\left||p_{k}^{\prime }\left(\ell \right)|\right|}$$ (24)

where $p_{k}\left(\ell \right)$ denotes the position vector of tendon *k* at s=ℓ and $p^{\prime }\left(s\right)$ denotes the derivative of the function p(s) with respect to *s*. Since the point of application of these forces act at a distance from the backbone, they produce a moment $M_{k}$ at the tip of the segment.

$$M_{k}=\left(p_{k}\left(\ell \right)-p\left(\ell \right)\right)\times F_{k}$$ (25)

The net force and moment acting on the backbone at the end of a segment is calculated by the vector summation of all tendon termination forces and moments. The distributed force, $f_{k}\left(s\right)$ is obtained by considering the internal force $n_{k}$, given by (Rucker and Webster, 2011a)

$$n_{k}^{\prime }\left(s\right)+f_{k}\left(s\right)=0.$$ (26)

If the tendon is assumed to be an inextensible string with no friction acting on it, the tension acting along it remains constant. While describing friction models is out of the scope of this paper, there are models that account for friction (Do et al., 2015; Back et al., 2016) for fully constrained tendon paths. Being perfectly flexible, it can only support tensions that are tangent to the tendon path. The internal force in the tendon is then given by,

$$n_{k}\left(s\right)=T_{k}\frac{p_{k}^{\prime }\left(s\right)}{\left||p_{k}^{\prime }\left(s\right)|\right|}.$$ (27)

Substituting the above in equation Eq. (26), gives $f_{k}\left(s\right)$ as

$$f_{k}\left(s\right)=T_{k}\frac{{\left[p_{k}^{\prime }\left(s\right)\right]}_{\times }^{2}}{||p_{k}^{\prime }\left(s\right)|{|}^{3}}.$$ (28)

These distributed forces also exert a distributed moment, $l_{k}\left(s\right)$ along the backbone

$$l_{k}\left(s\right)=\left(p_{k}\left(s\right)-p\left(s\right)\right)\times f_{k}\left(s\right).$$ (29)

Similar to the tendon termination forces, the net distributed force acting on the backbone is given by summing the individual forces.

A simplified approach would be to assume that the tendons apply only a pure moment at the point of termination (Gravagne et al., 2003a; Jones et al., 2009) or a force at the point of tendon termination resulting in an additional force and moment acting on the backbone (Dehghani and Moosavian, 2013a; Dalvand et al., 2018). While pure moment models result in good accuracy for in-plane bending, they do not predict nonplanar deformations resulting from external tip forces well (Rucker and Webster, 2011a). In order to model out-of-plane bending, an additional uniformly distributed tendon force, $f_{k}\left(s\right)$ described above can be considered to be acting on the backbone (Rucker and Webster, 2011a; Renda and Laschi, 2012; Hsiao and Mochiyama, 2017). Camarillo et al. (2008b) show that for a circular arc, considering tendon termination force $F_{k}$ and uniformly distributed force $f_{k}\left(s\right)$ is equivalent to only considering a pure moment acting on the backbone, described by Eq. (25).

#### 5.1.2 Forces due to Partially Constrained Tendon Path

**FIGURE 7:**
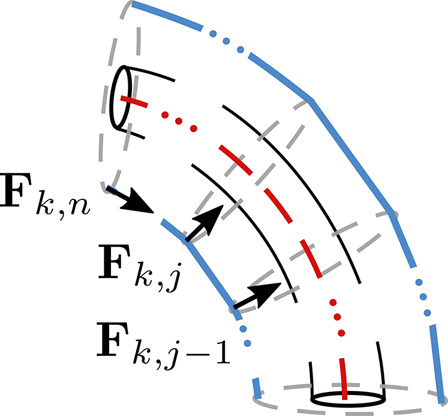
Diagrammatic representation of the forces $F_{k,j}$ applied by a discrete tendon path, where the portion between two disks is a line segment.

At every disk, the tendon *k* applies a force $T_{k,j}$ and a reaction force $T_{k,j+1}$, which together exert a moment on the backbone (shown in Figure 7). At the end of a segment, the tendon forces and moments acting on the *n*th disk are written as

$$F_{k,n}=T_{k,n}.\frac{\vec{P_{k,n}P_{k,n-1}}}{\left|\vec{P_{k,n}P_{k,n-1}}\right|}$$ (30)

$$M_{k,n}=\vec{O_{n}P_{k,n}}\times F_{k,n}$$ (31)

where $\vec{P_{1}P_{2}}$ represents a vector from $P_{1}$ to $P_{2}$.

The net force and moment acting on the backbone at the *j*th (j<n) disk by the *k* tendon are

$$F_{k,j}=T_{k,j}.\frac{\vec{P_{k,j}P_{k,j-1}}}{\left|\vec{P_{k,j}P_{k,j-1}}\right|}+T_{k,j+1}.\frac{\vec{P_{k,j}P_{k,j+1}}}{\left|\vec{P_{k,j}P_{k,j+1}}\right|}$$ (32)

$$M_{k,j}=\vec{O_{j}P_{k,j}}\times F_{k,j}.$$ (33)

When the friction between the tendons and disks can be neglected, the tendons passing through a disk can not transmit forces perpendicular to the disk. As a consequence, the component of the tendon force along this direction must be equal to zero.

However, in cases where friction cannot be ignored, an additional frictional force acting perpendicular to the disk needs to be included in the force balance equations. There are various tension propagation models (Jung et al., 2011; Kato et al., 2016; Gao et al., 2017; Yuan et al., 2019) that can be adapted for use in such situations.

#### 5.1.3 Extending to Multiple Segments

Actuating *g* segments results in more than one set of tendons passing through the first g−1 segments. The resulting tendon force and moment from each set of tendons can be calculated from the above equations. The net forces in a segment are given by the summation of the resulting forces from the tendons passing through that segment. Similarly, the net tendon termination forces can be obtained by adding the forces from each individual set of tendons.

### 5.2 Constitutive and Equilibrium Equations

As the robot backbone undergoes elastic deformations while experiencing small strains (Gravagne et al., 2003b), Hooke’s law is used to develop the corresponding static models. The backbone is modelled as a beam, undergoing linear elastic deformation where the stress experienced by the backbone is linearly proportional to the strain. In addition, we assume that the backbone and tendons are inextensible. In general, the undeformed reference position of the backbone can be represented using the variable curvature representation as v*(s) and u*(s). The angular and position deformations are then given by (v(s)−v*(s)) and (u(s)−u*(s)). Using Hooke’s law, the internal force and moment vectors, n(s) and m(s) obey the following constitutive equations

$$n\left(s\right)=K_{SE}\left(v\left(s\right)-v\text{*}\left(s\right)\right)$$ (34)

$$m\left(s\right)=K_{BT}\left(u\left(s\right)-u\text{*}\left(s\right)\right)$$ (35)

where

$$K_{BT}=\left[\begin{array}{ccc} EI_{xx}\left(s\right) & 0 & 0\\ 0 & EI_{yy}\left(s\right) & 0\\ 0 & 0 & GJ \end{array}\right]$$ (36)

$$K_{SE}=\left[\begin{array}{ccc} GA\left(s\right) & 0 & 0\\ 0 & GA\left(s\right) & 0\\ 0 & 0 & EA\left(s\right) \end{array}\right]$$ (37)

where *A* is the area of cross section as a function of *s*, *G* is the shear modulus, $I_{xx}$ and $I_{yy}$ are the second area moments and *J* is the polar second moment of cross sectional area.

#### 5.2.1 Variable Curvature

The Cosserat theory of elastic rods (CRT) uses the variable curvature representation to assign six degrees of freedom to each point on the backbone (three translational and three rotational) (Altenbach et al., 2013). It can account for shear deformations as well. For a rod in static equilibrium, the internal force and moment vectors, n(s) and m(s) obey

$$\frac{\text{d}n\left(s\right)}{\text{d}s}+\overset{m}{\underset{k=1}{\sum }}f_{k}\left(s\right)=0$$ (38)

$$\frac{\text{d}m\left(s\right)}{\text{d}s}+\frac{\text{d}p\left(s\right)}{\text{d}s}\times n\left(s\right)+\overset{m}{\underset{k=1}{\sum }}l_{k}\left(s\right)=0$$ (39)

where f(s) and l(s) are the externally distributed forces and moments. Uniformly distributed force given by Eq. (28) and gravity are included in the expression for f(s). The Hooke’s law equations, defined in Eq. (34) and (35) are used to couple the deformations given by Eq. (3) and (2) with the internal forces and moments.

The tendon termination and external forces and moments are included in the boundary conditions. The resulting changes in internal force and moment due to these forces at the end of a segment is given by

$$n\left(\ell^{-}\right)=n\left(\ell^{+}\right)+\overset{m}{\underset{k=1}{\sum }}F_{k}+F_{ext}$$ (40)

$$m\left(\ell^{-}\right)=m\left(\ell^{+}\right)+\overset{m}{\underset{k=1}{\sum }}M_{k}$$ (41)

where $\ell^{-}$ indicates locations just before the point of termination at s=ℓ. Jones et al. propose a 3D static model using CRT for continuum robots (Jones et al., 2009). Dehghani and Moosavian (2011) present a simplified 2D Cosserat model for continuum arms. Renda and Laschi (2012) and Rucker and Webster (2011a) couple the tendon behaviour into the model equations for the three dimensional case.

#### 5.2.2 Constant Curvature

For backbones assumed to be bending with constant curvature, Euler Bernoulli beam theory can be applied. It assumes that the effects of shear and twist are negligible (Bauchau and Craig, 2009). The bending moment along the beam is assumed to be proportional to its curvature. Hooke’s law reduces to a one-dimensional equation and is given by

m=κEI. (42)

It has been used to model both segments (Camarillo et al., 2008b; Ryu et al., 2019) and subsegments (Yuan and Li, 2018; Yuan et al., 2019; Rone and Ben-Tzvi, 2014a; Dehghani and Moosavian, 2013a; Dehghani and Moosavian, 2013b; Dehghani and Moosavian, 2014a; Dehghani and Moosavian, 2014b). To account for bending and twist with CC_sub_ the components of curvature of a subsegment *j* along the three axis are used to represent the 3D circular arc. Expressed with respect to $R_{j}$, the constitutive equation for subsegment *j* is simplified from Eq. (34) and expressed as

$$m_{j}=R_{z}\left(\phi \right)R_{y}\left(\theta_{j}\right)\left[\begin{array}{ccc} EI_{xx} & 0 & 0\\ 0 & EI_{yy} & 0\\ 0 & 0 & GJ/\ell_{j} \end{array}\right]\left[\begin{array}{c} 0\\ \kappa_{j}\\ \epsilon_{j} \end{array}\right]$$ (43)

where $\epsilon_{j}$ represents the twist about the $z_{j}$ axis while $\kappa_{j}$ is the curvature of the subsegment *j*. As the torsion and bending is decoupled, the section is modelled using St. Vernant’s torsion theory where the twist angle is assumed to vary linearly along the section where constant moment is applicable. The net force and moment acting on the distal subsegment *n* is given by (Yuan et al., 2019)

$$F_{n}=\overset{m}{\underset{k=1}{\sum }}F_{k,n}+F_{ext}$$ (44)

$$M_{n}=\overset{m}{\underset{k=1}{\sum }}M_{k,n}+\vec{O_{n-1}O_{n}}\times F_{n}$$ (45)

where $M_{k,n}$ is the moment due to tension defined in Eq. (31). For a subsegment *j*, the net force and moment acting in the subsegment is given by the recursive equation

$$F_{j}=\overset{m}{\underset{k=1}{\sum }}F_{k,j}+F_{j+1}$$ (46)

$$M_{j}=\overset{m}{\underset{k=1}{\sum }}M_{k,j}+\vec{O_{j-1}O_{j}}\times F_{j}+M_{j+1}.$$ (47)

where $F_{j+1}$ and $M_{j+1}$ are the forces and moments propagated from the succeeding subsegments.

#### 5.2.3 Pseudo Rigid Body (PRB) Models

The 3 R PRB model proposed by Su (2009) has been used to propose a model that can account for twists in each subsegment. As detailed in Section 3, the backbone is approximated by 4 rigid links connected by 3 torsion springs. The bending components of the net moment $M_{j,q}$ in each spring q=1,2,3 is proportional to $\theta_{j,q}$, which is written as

$$M_{j,q}.y_{j}=k_{q}\theta_{j,q}$$ (48)

$$k_{q}=K_{{\text{Θ}}_{q}}\frac{EI}{\ell }.$$ (49)

The values of the characteristic parameters, γ and $K_{\text{Θ}}$ are optimized by Chen et al. (2011) to maximize the PRB model accuracy for one section subject to tip moments and forces and presented in Table 1.

**TABLE 1:** Optimized values of characteristic parameters.

$\gamma_{0}$	$\gamma_{1}$	$\gamma_{2}$	$\gamma_{3}$	K_Θ_1__	K_Θ_2__	K_Θ_3__
0.125	0.35	0.388	0.136	3.25	2.84	2.95

The static model is then obtained by writing the equilibrium conditions of the TDCR at each joint. The point at the center of joint *q* belonging to subsegment *j* is called $O_{j,q}$. Considering the same tendon forces $F_{j}$ as for the constant curvature model, given by Eq. (44) and (46), the net moments applied at joint *q* along the last subsegment and for j<n are written as

$$M_{n,q}=\overset{m}{\underset{k=1}{\sum }}M_{k,n}+\vec{O_{n,q}O_{n}}\times F_{n}$$ (50)

$$M_{j,q}=\overset{m}{\underset{k=1}{\sum }}M_{k,j}+\vec{O_{j,q}O_{j}}\times F_{j}+M_{j+1}.$$ (51)

In order to compute the bending plane angle $\phi_{j}$, Huang et al. (2019) propose a geometrical relation in the case the robot is composed of one sub-segment and is not subject to twist. In this scenario, the component $M_{j}.z_{j}$ is zero and $\phi_{j}$ can be determined so that $M_{j}$ is collinear with the normal of the bending plane $y_{j}$ using the relation

$$\left\|M_{j}\times y_{j}\right\|=0.$$ (52)

To the best of our knowledge, this model has not been extended to TDCR with arbitrary number of segments or sub-segments and subject to twist, under the influence of an external force. In this case, the component $M_{j}.z_{j}$ is not zero, and Eq. (52) cannot be enforced anymore. We realize this extension by first considering that the angle $\phi_{j}$ is due to the subsegment bending only. As a result, we write Eq. (52) with $M_{j}^{⋆}$ instead of $M_{j}$ where

$$M_{j}^{⋆}=M_{j}-M_{j}.z_{j}.$$ (53)

Second, we add an additional equilibrium equation to account for the twist, by calculating the net moment in with respect to the frame $R_{j}$.

$$GJ\epsilon_{j}=\ell_{j}\left(T_{j-1}^{j}M_{j}\right).z$$ (54)

where $M_{j}$ is computed using Eq. (45) for j=n and Eq. (47) for j<n. In conclusion, equations Eq. (48), (52), and (54) constitutes the extended PRB model.

## 6 Comparison of TDCR Models and Guidelines

### 6.1 Synthesis

**FIGURE 8:**
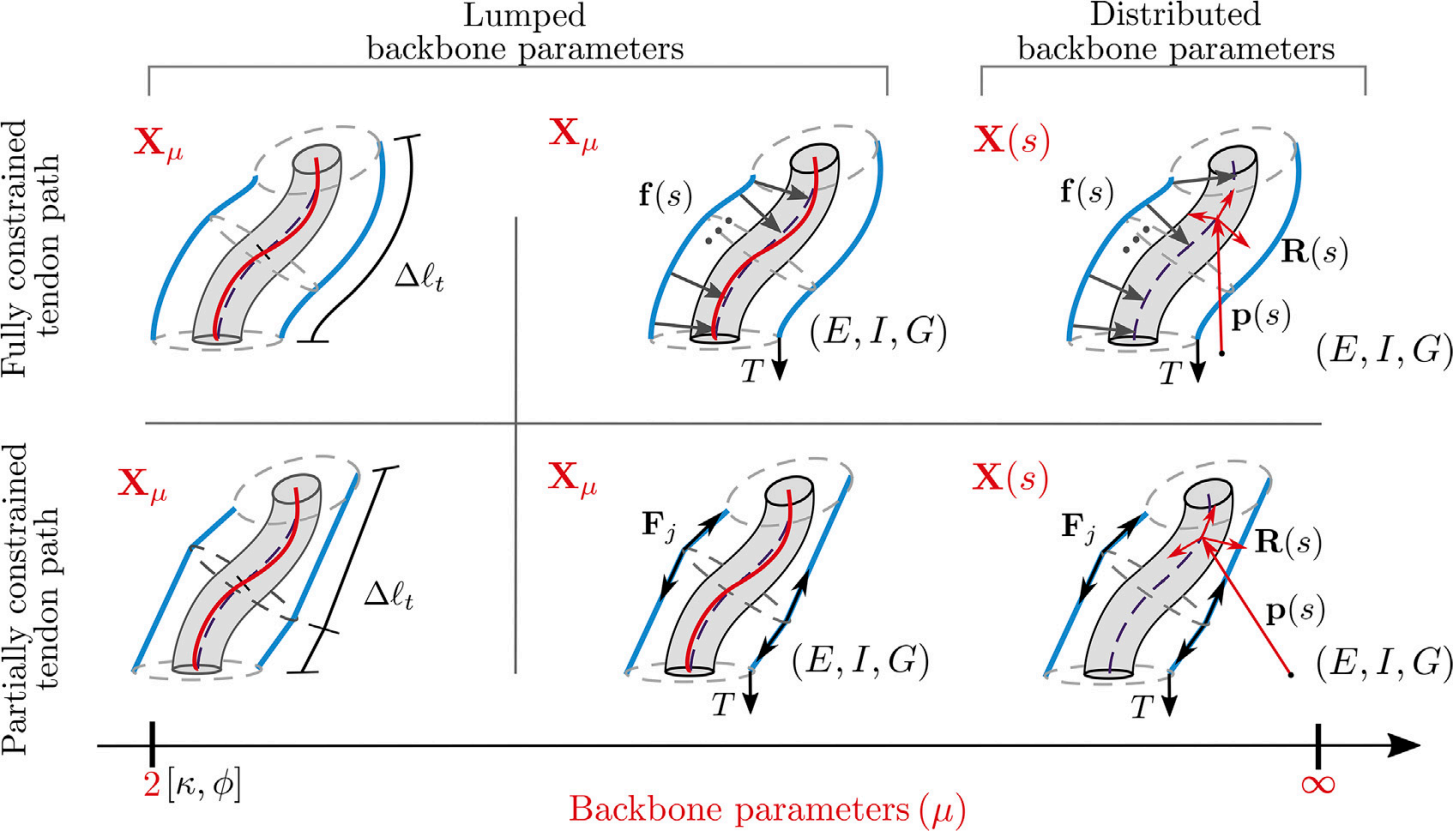
Proposed classification of different modelling approaches based on the backbone parameters required to define the backbone and assumed tendon path. The left column shows pure kinematic modelling, which maps the tendon lengths to the backbone pose while the statics modelling in the right column considers the backbone properties such as Young’s modulus (*E*), moment of inertia (*I*), shear modulus (*G*) as well as the effect of resulting tendon forces (f(s) or $F_{j}$) acting on it.

As reflected in the previous sections, modelling the kinematics or the statics of TDCR can be decomposed into two tasks: choosing between lumped or distributed parameters to represent the backbone and assuming the tendon path is either fully or partially constrained. We classify the modelling approaches accordingly. The classification is summarized in Figure 8. The kinematic models (left column) are used for modelling TDCRs, where the segment is assumed to obey the CC assumption. The robot shape can thus be described with a minimal number of parameters. The tendon tensions and external forces (right column) lead to a variation in the backbone curvature, and is represented with a higher number of parameters.

Choosing a suitable model for a desired TDCR design and application is not trivial. Distributed parameters can accurately describe the variation of curvature in the presence of external forces, but the resulting model is computationally demanding due to the required theoretically infinite number of parameters. The accuracy of lumped parameters depends on the deformation of the robot under external forces, which in turn depends on the magnitude of these forces, the robot length, and stiffness. In cases that require a large number of parameters to obtain sufficient accuracy, lumped parameterization can become more computationally expensive than distributed parameterization (Barrientos-Diez et al., 2021). Regarding the tendon path assumption, a naive strategy would be to consider partially and fully constrained tendons when they are guided using spacer disks and through a lumen in the backbone, respectively. However, a static model considering fully constrained tendons is proved to be accurate in predicting the shape of a TDCR composed of spacer disks in Rucker and Webster (2011a). Similarly, Norouzi-Ghazbi and Janabi-Sharifi (2020) use the partially constrained tendon path assumption to model a TDCR whose tendons are guided through lumen.

### 6.2 Scenario

To provide guidelines for choosing a suitable model of TDCR, we implement the most common backbone representations and tendon assumptions in the literature and compare their performances in terms of accuracy and computation speed. The corresponding models are listed in Table 2. We consider a purely kinematic model for the CC assumption which considers that the curvature of each segment of the robot is constant. It has been extensively used due to its simplicity and computational efficiency as explained by Webster and Jones (2010). While there have been closed form solutions using kinematic approaches (Gravagne and Walker, 2000c), they do not consider the backbone material properties and cannot be used for the case where an external force (e.g. gravity, weight of a tool placed at the tip, external contact) acts on the robot body, highlighting the need for consideration of other models.

**TABLE 2:** Models of TDCR used in the case study. Backbone representations CC: Constant curvature, CC_sub_: Constant curvature assumption applied to each sub segment, PRB: Pseudo rigid body, VC: Variable curvature. Tendon assumptions FC: Fully constrained, PC: Partially constrained.

Compared models									
Model	Backbone representation	Tendon assumption	Inputs	Backbone parameters	Equations	Reference
CC	CC	FC	$l_{1},\ldots ,l_{4}$	$X_{\mu }=\left(\kappa_{1},\phi_{1},\kappa_{2},\phi_{2}\right)$	(8,16–18)	Webster and Jones (2010)
CC_sub_	CC	PC	$T_{1},\ldots ,T_{4}$	$X_{\mu }=\left(\kappa_{j},\phi_{j},\epsilon_{j}\right)$	(8,30–33,43–47)	Yuan et al. (2019)
				j=1...2n		
PRB	PRB	PC	$T_{1},\ldots ,T_{4}$	$X_{\mu }=\left(\theta_{1j},\theta_{2j},\theta_{3j},\phi_{j},\epsilon_{j}\right)$	(12,30–33,48–54)	-
				j=1...2n		
VC	VC	FC	$T_{1},\ldots ,T_{4}$	X(s)=(u,v)(s)	(2–3,28–29,38–41)	Rucker and Webster, 2011a
Reference model						
VCref	VC	PC	$T_{1},\ldots ,T_{4}$	X(s)=(u,v)(s)	(2–3,30–33,38–41)	Gao et al. (2017)

The CC_sub_ model is a static model which considers the backbone curvature to be constant along each subsegment. The PRB and VC models are static models as well that consider pseudo rigid body and variable curvature representations respectively. We consider especially the extended PRB model including torsion. The static models have been used to model the impact of various loading conditions on the shape of TDCR (Rucker and Webster, 2011a; Huang et al., 2019; Yuan et al., 2019). Their performances are compared for a relevant design of TDCR with different design parameters and in two relevant scenarios.

We consider a TDCR composed of two segments of length *L*, each segment being actuated by antagonistic pairs of tendons. We choose spacer disks to guide the tendons along the backbone, in order to reproduce the behaviour of both partially and fully constrained tendons. Indeed, the tendons are normally partially constrained along the robot backbone, and when the number of disks become large and tend to infinity, they can be assumed to be fully constrained. Each segment has *n* spacer disks. We assume that the weights of the disks and backbone are negligible and there are no frictional forces. The effects of the assumption on the tendon’s constraint and of the backbone representation are studied considering two scenarios, respectively. In the first, we consider that the robot is subject to tendon forces only and we vary the number of sub-segments *n*. For the sake of simplicity, we only consider configurations where the robot bends in a plane. Therefore, each segment is actuated with a single antagonistic pair of tendons, so that the robot bends in the xz plane. Tendons (1,2) and (3,4) actuate the proximal and distal segment respectively. In the second scenario, we consider the same arrangement of tendons but consider in addition that the robot is subject to planar tip forces, along x, and transverse ones, along y. The CC model is excluded from this scenario since it cannot account for external forces. The impact of these forces on the robot shape depends on the bending stiffness of the backbone. The models are thus compared in these two loading cases for multiple values of stiffness.

For assessing accuracy, we compare each model’s results with a reference model that is the most general in modelling a TDCR with spacer disks, making as little simplifying assumptions as possible. Thus, we employ a model that accounts for partially constrained tendons, does not make simplifying assumptions on the backbone geometry and uses the distributed parameterization. Consequently, the model uses variable curvature representation. We consider each subsegment as a rod experiencing tip forces and moments due to tendon interaction at each disk’s location, as done in Gao et al. (2017). Doing so allows us to model the behaviour of the partially constrained tendon path resulting from the use of spacer disks. As we use the VC backbone representation, we label this reference model as VC_ref_. It uses CRT to obtain the equilibrium equations with boundary conditions at each disk given by Eq. (40), (41). The resulting forces and moments are calculated using Eq. (44) and (47). Its characteristics are listed in Table 2.

## 7 Method

The accuracy of a model is estimated for a given workspace of the robot. The workspace is defined here in terms of the angular displacement of the robot tip during tendon actuation. In the literature, angular displacements of up to ±90 degrees are classically considered (Rucker and Webster, 2011a; Huang et al., 2018; Yuan and Li, 2018). The accuracy assessment is then performed with the following steps.

First, the actuation space required to achieve the desired workspace is calculated. The maximum tendon force $T_{M}$ required to achieve an angular displacement of $\theta_{M}=\pm 90$ degrees is estimated by considering the tendons to be fully constrained and to apply a point moment at the tip of each section. In this case, the maximum tip angular displacement $\theta_{M}$ is obtained when $T_{M}$ is applied to tendons 1 and 3 and is written as

$$\theta_{M}=\frac{3r_{d}T_{M}L}{EI}$$ (55)

Therefore, $T_{M}$ is computed using Eq. (55). A set of tendon forces is then picked from the actuation space. The difference between the models is observed to increase with the actuation forces. Therefore, we consider that tendon forces can be either equal to 0 or to $T_{M}$. With the 4 tendons of the considered TDCR, this gives a set of N=16 sets of actuation inputs.

The different models are then solved for each set of actuation inputs. The CC_sub_, PRB, VC and VC_ref_ are solved using numerical solvers introduced in the Section 6.4. The CC model requires the tendon forces to be converted to the resulting tendon displacements for it to be solved. To do this conversion, the robot shape is computed with the VC model and the corresponding tendon length is deduced from the path followed by the tendons along the backbone (Oliver-Butler et al., 2019). The length of tendon *k* is written as

$$l_{k}={\int^{\text{​}}}_{s=0}^{2L}|p_{k}^{\prime }s|ds$$ (56)

**FIGURE 9:**
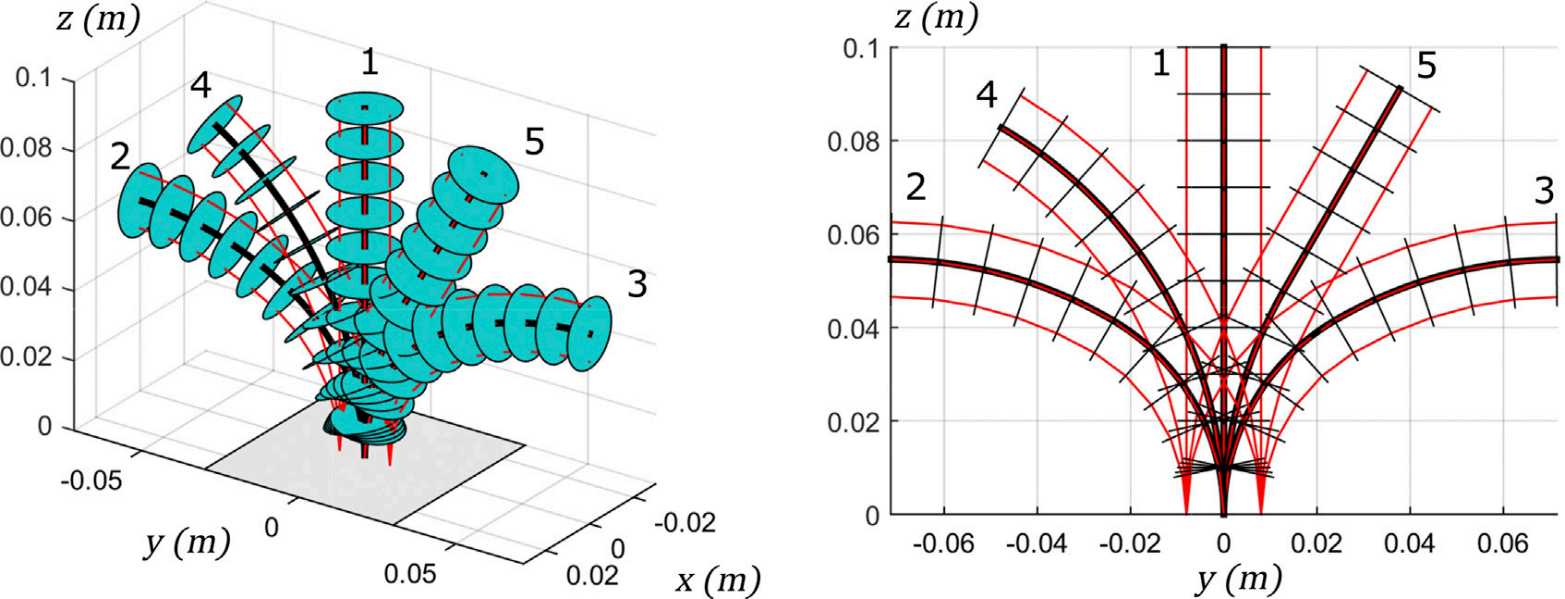
Subset of the TDCR configurations considered in the model comparison, for *n* = 5 and without external forces at the tip. Left: 3D view, Right: Planar view. Maximum or zero tendon tension is applied to each tendon for the two segments. $\left[T_{1},T_{2},T_{3},T_{4}\right]=$ 1:[0,0,0,0], 2: $\left[T_{M},0,T_{M},0\right]$, 3: $\left[0,T_{M},0,T_{M}\right]$, 4: $\left[{\mathrm{0,0,T}}_{M},0\right]$, 5: $\left[0,T_{M},0,0\right]$. The backbone is represented in black, the spacer disks in blue and the tendons in red.

Solving the models results in 16 robot shapes per model, a subset of which is presented for the sake of clarity in Figure 9.

Finally, the robot shapes are compared to the results given by VC_ref_. We propose the use of two metrics, denoted by $e_{P}$ and $e_{R}$, to measure the differences in tip position and orientation respectively. Let $p_{q}$ and $R_{q}$ be the tip position and orientation for the *q*th configuration computed with one of the four model, and $\left(p_{\text{ref},q},R_{\text{ref},q}\right)$ the tip pose obtained with the reference model.

$$\begin{array}{l} e_{P}=\frac{1}{N}\overset{N}{\underset{q=1}{\sum }}\frac{\left|\left|p_{q}-p_{\text{ref},q}\right|\right|}{2L}\\ e_{R}=\frac{1}{N}\overset{N}{\underset{q=1}{\sum }}{\text{cos}}^{-1}\left(\frac{\text{trace}\left(R_{q}R_{\text{ref},q}^{T}\right)-1}{2}\right) \end{array}$$ (57)

The metric $e_{P}$ is the mean distance of the robot tip to the reference model over the workspace, and is expressed as a percentage of the robot length. The metric $e_{R}$ is the mean angle between the robot tip directions over the workspace, considering axis-angle representation of the orientations.

The computation time and the stability of the numerical solver are strongly dependant on the provided initial guess. Therefore, in order to perform a fair comparison in terms of computation time, similar initial guesses must be used. However, it is not possible to find a common initial guess which allows for all the models to converge for every picked configuration in the workspace. Consequently, we evaluate the computation time needed to compute one configuration only, which is configuration 2 in Figure 9. The initial guess for all the models is the robot shape without tendon or external forces, which is configuration 1 in the Figure. Each model is solved 100 times in order to filter eventual fluctuations of computation time. The values of computation time presented here are the mean values over the 100 solutions.

### 7.1 Implementation

We consider the modelling of a TDCR, composed of a flexible backbone and spacer disks. The robot parameters are provided in Table 3. Since we assume a frictionless system, we enforce the component of the tendon force perpendicular to the disk to be zero by computing the tendon force at each disk using the following relation

$$F_{k,j}^{⋆}=F_{k,j}-F_{k,j}.z_{j}.$$ (58)

**TABLE 3:** Parameters of the TDCR used for the case study.

Parameter	Value
Segment length *L* (m)	$50*10^{-3}$
Disk radius $r_{d}$ (m)	$8*10^{-3}$
Backbone diameter (m)	$0.8*10^{-3}$
Young modulus *E* (Pa)	$2.11*10^{11}$
Quadratic moment *I* (m4)	$2.01*10^{-14}$
Stiffness EI (N.m2)	$4.2*10^{-3}$
Position of tendons in ${\left(R\right)}_{j}$ frame	
$\vec{O_{j}P_{j,1}}$, $\vec{O_{j}P_{j,3}}$	$\left[\begin{array}{ccc} 0 & r_{d} & 0 \end{array}\right]$
$\vec{O_{j}P_{j,2}}$, $\vec{O_{j}P_{j,4}}$	$\left[\begin{array}{ccc} 0 & -r_{d} & 0 \end{array}\right]$

The models of TDCR and their resolution are implemented in C++ language as well as in Matlab 2015a (Mathworks Inc.). The source codes for each language are provided with this paper. In the C++ implementation, the non-linear equations composing the CC_sub_ and PRB models are solved using the Levenberg-Marquardt algorithm implemented in the GNU GSL library. The resulting backbone shape is computed with Eq. (8) and (12) respectively. VC and VC_ref_ models consist in differential equations with initial and final boundary conditions and are therefore solved with a shooting method (Rucker and Webster, 2011a). In this method, the initial boundary conditions are guessed and the differential equations are integrated until the difference between the state at the robot tip and the desired final boundary condition vanishes. For the VC model, the final boundary conditions are expressed as the force and moment equilibrium at the tip of the robot, while the initial values of u and v at the robot’s proximal end are guessed, resulting in six non-linear equations to solve. For the VC_ref_ model, the boundary conditions include the force and moment equilibrium at each disk, to account for the discrete forces applied by the tendons, while the initial values of u and v are guessed at the beginning of each subsegment, resulting in 2n(6) equations to solve. The Runge-Kutta-Fehlberg (4,5) algorithm is used to integrate the differential equations and the Levenberg-Marquardt algorithm is used to solve for the initial conditions. We use also the Eigen library (http://eigen.tuxfamily.org) for efficient matrix and vector calculations. The C++ model implementations are solved on an Intel Core i5-9600 CPU running at 3.10 GHz.

In the MATLAB implementation, the CC_sub_, PRB and the shooting method are solved using the Trust-region-doggleg algorithm implemented in the function fsolve. The integration of the differential equations is performed using the 4th order Runge-Kutta method implemented in the ode45 solver.

## 8 Results and Guidelines

### 8.1 On the Number of Disks

**FIGURE 10:**
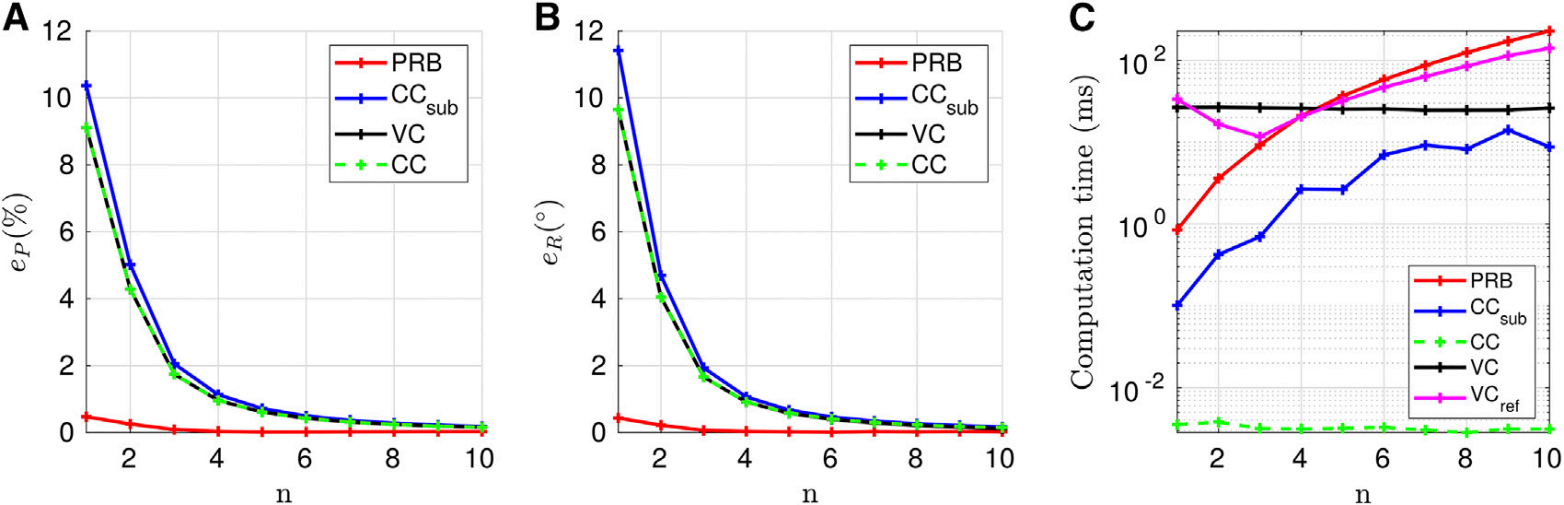
Evolution of the models accuracy according to the number of disks per segment. The metrics $e_{p}$ and $e_{R}$ represent the deviation of the tip position and orientation respectively.

The model error values obtained for the first scenario are presented in Figures 10A,B. We observe that the number of disks has a significant effect on the robot. The tip error between the VC models with fully and partially constrained tendon reaches 9.12% with respect to the robot length for n=1. It then decreases as *n* increases, and reaches 0.16% for n=10. This last error is consistent with the low tip error obtained in , where a TDCR with 12 spacer disks is accurately modelled considering fully constrained tendons. Note that the results of the VC model do not Rucker and Webster, 2011a depend on *n* since the tendons are fully constrained, while the result of the VC_ref_ model does. The CC model gives identical results as the VC model, which is expected since considering fully constrained tendons without any external force leads to a robot with constant curvature (Camarillo et al., 2008b).

The CC_sub_ shows a similar pattern, even though it considers the partially constrained tendons of the considered TDCR. This can be explained by the fact that the robot is deformed by moments due to the distance between the tendons and the backbone, but also by tendon forces. The lower number of disks results in longer subsegments that experience the same magnitude of tendon forces, resulting in subsegments with non-constant curvature that are not accounted for by the model. The PRB model is the most accurate in the present case study, with its error being at most 0.47% and 0.43° for n=1. Its ability to account for variable curvature along a subsegment and the optimised parameters γ and $K_{\text{Θ}}$ (Chen et al., 2011) for planar deformation of a beam subject to tip moments and forces contribute towards its high accuracy.

The evolution of the computation time with respect to *n* is presented in Figure 10C. The CC model is the fastest as it requires the lowest number of backbone parameters to compute and has an analytical solution. Its computation time is constant and equals 2.7 µs. The PRB and CC_sub_ models present a similar tendency in terms of speed. As the number of disks increases and, consequently, the number of subsegments, the number of unknowns increases as well, resulting in higher computation times. For n>=4, the PRB model becomes more cumbersome, compared to the VC model. For the CC_sub_ model, this phenomenon happens for a larger number of disks, since only 3 backbone parameters per subsegment must be computed instead of 5 for the extended PRB. The computation time of the CC_sub_ model is evaluated for higher values of *n*, and reveals to be larger than the VC model for n=13. The VC_ref_ model provide similar computation time compared to the PRB model for n>=3. The higher computation time for n<3 may be due to the relatively large length of the subsegments. In this case, the variation of curvature due to tendon forces are more important, resulting in a robot shape more different from the initial guess.

From these results, we deduce the following guidelines. When considering a TDCR with spacer disks, the VC_ref_ and PRB model are the best choice due to their higher accuracy. They are particularly interesting for lower number of disks where they are also computationally efficient. For n>=5, all the other models have errors below 1% and 1°. In that case, the pure kinematic CC model seems to be the best choice in this scenario where the TDCR is not subjected to external forces.

It is as accurate as the other models for any number of disks, and is substantially faster. In cases where a static model is required due to the presence of external forces and for 5<=n<=13, the CC_sub_ model can be chosen since it is the second faster model. For n>13, the VC model would be the best choice since it provides the same accuracy than the other approaches and is faster.

### 8.2 On the Backbone Stiffness

**FIGURE 11:**
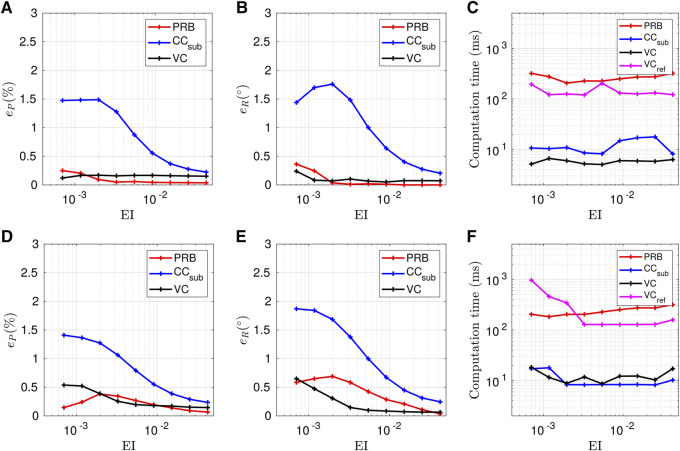
Evolution of the models accuracy according to the backbone stiffness when considering a planar **(A–C)** and transverse **(D–F)** tip force for *n* = 10. The metrics $e_{p}$ and $e_{R}$ represent the deviation of the tip position and orientation respectively.

The model error values obtained for the second scenario as a function of the backbone stiffness are presented in Figure 11. The number of disks per segment is fixed at n=10. The stiffness is varied with a logarithmic scale in the interval $\left[10^{-1}EI,10EI\right]$. The planar and transverse forces applied at the robot tip are $F_{ext}={\left[\begin{array}{ccc} 0 & 0.5 & 0 \end{array}\right]}^{T}$ N and $F_{ext}={\left[\begin{array}{ccc} 0.5 & 0 & 0 \end{array}\right]}^{T}$ N respectively.

When an external tip force is applied on the TDCR, Figures 11A,B,D,E show that PRB and VC models provide results close to the reference and that the errors decrease globally as the backbone stiffness increases. For higher backbone stiffness, a larger magnitude of tendon force is required to maintain the same deflection angle. PRB and VC models give better accuracy than CC_sub_, especially for lower values of stiffness. In these cases, the tip force has a larger impact on the backbone shape than the bending moment due to the tendons. As a result, the curvature is not constant along the subsegments. This variation of curvature in the subsegment leads to errors up to 1.5% and 1.87°, as shown in Figures 11D,E.

The PRB model is particularly accurate for planar forces, with minimum position and orientation errors of 0.04% and 0.01° respectively, which is consistent since its parameters have been optimized for this scenario. On the other hand, it does not perform as well for non-planar deformations caused by transverse tip forces, especially in terms of tip orientation as shown in Figure 11E.

The evolution of the computation times for the two loading cases are presented in Figures 11C,F. Problems of convergence were encountered when considering the maximum tendon force $T_{M}$ and the tip force $F_{ext}$. Therefore, for all the models to converge starting from the same initial guesses, the computation time is evaluated for a tendon force and a tip force of $T_{M}/2$ and $F_{ext}/2$ respectively. For the two loading scenarios, the speed of CC_sub_ and VC model is approximately constant and is one order of magnitude lower than the PRB and VC_ref_ models. For the PRB model, the computation time increases slightly as the backbone stiffness increases. The maximum computation time obtained for the VC and VC_ref_ models when transverse forces are applied is higher than the values obtained for planar forces. This increase is to be expected, since transverse forces induce torsion of the backbone. Interestingly, the computation time of the CC_sub_ model stays equal to approximately 10 ms in both loading scenarios.

It is especially higher than the computation time of the VC model for planar tip forces, even though it was the opposite in the first scenario. This is attributed to the convergence of the CC_sub_ and VC models which depends on the mixed tendon and external forces applied on the robot in a non-trivial manner.

From these results, we deduce the following guidelines for the case of a TDCR, subject to a tip force: In applications where model accuracy is the primary goal and planar forces are applied to the robot, the VC_ref_ and PRB models seem to be the best choice. In the case of transverse force, the VC and VC_ref_ models are the most accurate. If the computation time is also a concern, the VC model seems the most suitable in both cases. The CC_sub_ model can also be used for stiffer backbones.

## 9 Discussion

In this paper, we present the existing models of TDCR using a common formalism and compare these models for the first time on a typical design of TDCR. Comparing the robot shape obtained with each model allows us to formulate guidelines for modelling a TDCR according to its design. However, finding a unified formalism poses challenges as different terminology is used in the literature to describe various models. Also, care must be taken while comparing and interpreting the results as each model is developed and evaluated for different applications and robot design. In this section, we discuss some of these difficulties to provide the reader with a better understanding of the results.

### 9.1 Expression of the Tendon Forces at the Disks

Writing the models with a common formalism raises two questions that have not been fully answered yet.

First, the tendon forces exerted on each disk in the case of partially constrained tendons are not expressed with the same equations in the literature. Two forces are considered to be applied at the point $P_{k,j}$ of disk *j*, pointing towards $P_{k,j-1}$ and $P_{k,j+1}$ respectively, and the net moments and forces propagated from the preceding subsegments are considered. However, in the PRB models of Huang et al. (2018); Huang et al. (2019), each tendon is assumed to apply only one force in the direction of $\vec{P_{k,j}P_{k,j-1}}$, and the equilibrium equations are written without considering the propagated moments and forces from the preceding subsegments. Simulations show that these two expressions lead to the same robot shape when no external forces or moments are applied. However, the results vary when an external force is applied at the tip. A thorough investigation of these different tendon force expressions is required to understand the assumptions for which they are valid.

Second, the question of the component of tendon forces transmitted to the backbone through the first n−1 disks, in the absence of friction has not been discussed in the literature. In this paper, we consider that no force can be generated in the direction perpendicular to the disk for the frictionless case, and we enforce that by using Eq. (58). In the literature, the expression for tendon force Eq. (33) is used without enforcing that the component perpendicular to the disk to be zero. This component is zero at disk *j* when the bending angles of sub-segments *j* and j−1 are equal. This equality implies that the backbone curvature must be constant along the segment, which is only true when no tip force is applied. If a force is applied at the robot tip, the curvature is not constant anymore, and the resulting tendon forces have a component perpendicular to the disk. This behaviour has been confirmed in simulations. The models result in visibly different robot shapes depending on whether Eq. (58) is enforced. This phenomenon should then receive attention, especially while evaluating the accuracy of TDCR models using experimental data.

### 9.2 Comparison of TDCR Models

We discuss three major points regarding the results presented in the comparison of TDCR models.

First of all, the behaviour of obtained errors in the case study depends on the considered TDCR design. This paper considers a TDCR with spacer disks and provides guidelines according to the number of disk per segment and the backbone stiffness. While we do not consider the case of a TDCR with fully constrained tendons passing through lumens along the backbone, the provided case study can still be adapted to provide guidelines for the same. For a fully constrained tendon path, the VC model becomes the most general model and can be then be considered the reference. As a result, for the load-free scenario (see Figure 10), we can say that the robot shape can be obtained accurately and with low computation time with the CC_sub_ and PRB model by dividing each segment into a minimum of n=3 subsegments. In the case of planar and transverse forces, the PRB model would be the most accurate.

Second, the errors are computed with respect to a reference model, and each model is for the same set of robot parameters. The CC, CC_sub_, PRB and VC model can be made more accurate by individually calibrating each model’s parameters using experimental data. This calibration step is expected to give different robot parameters for each model, since they do not consider the same assumptions. Thus, we will obtain different design parameters that provide higher accuracy for a given prototype of TDCR. This calibration will allow us to account for modelling inaccuracies to some extent. For example, in Rucker and Webster, 2011a, a higher backbone stiffness than the nominal value provided by the manufacturer is obtained after calibration, which may be due to the assumption of fully constrained tendons or the presence of friction. This individual calibration has not been done in our case studies for a fair comparison and easy interpretability of results. Consequently, the reported errors can be viewed as upper bounds, with the possibility of reducing them through calibration.

Third, the values of computation time depends on the chosen method of implementation. We believe the C++ model implementations can be further optimized to increase computation speed. Moreover, the computation time also depends on the initial guess. The closer the initial guess from the desired solution, the faster the resolution of the model. In a practical scenario of deployment or trajectory tracking, where the robot tip must pass through several points $p_{i}$, there is usually no significant difference between two successive robot shapes. As a result, the TDCR shape at point $p_{i}$ can thus be computed in a shorter time by solving the model with the shape at point $p_{i-1}$ as an initial guess. The computation times reported in this paper are calculated using the same initial guess of an undeformed backbone. The difference between this initial guess and the final deformed shape is the largest possible case, in the sense that considering any other initial guess with a larger deviation might lead to numerical convergence issues. Consequently, the reported errors can also be seen as upper bounds, with the possibility of reducing them by choosing an initial guess closer to the actual solution.

### 9.3 Relation Between TDCR Designs and Models

In summary, the comparative case studies show that a given design of TDCR can be modelled by considering different assumptions on the tendon path and backbone representations. The graphs not only compare the performances of some of the more common models for a TDCR design with spacer disks, but can also be used to study their performances for a design with lumen by comparing the results of each model to that of the VC model instead of VC_ref_.

**FIGURE 12:**
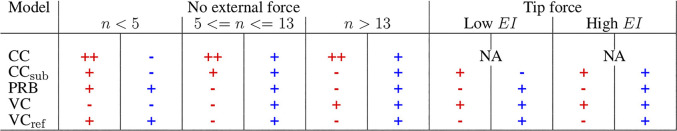
Advantages (+) and limitations (−) of each model considered in the case study, in terms of accuracy (blue) and computation time (red), according to the design parameters of a TDCR composed of spacer disks. Forces and limitations for TDCR using lumen to guide the tendons can be obtained by considering the same table but reversing the accuracy ”+” and ”−” signs of the column *n* < 5. NA: Not applicable.

The forces and limitations of each model are summarized in Figure 12. The models have different performances in terms of accuracy and computation time depending on the loading scenarios and robot design. One interesting observation is that it is not necessary for the tendon path assumption to reflect the actual TDCR design, confirming what was already proposed by Rucker and Webster, 2011a; Norouzi-Ghazbi and Janabi-Sharifi (2020); Barrientos-Diez et al. (2021). Models assuming fully constrained tendons, such as the VC or CC models, can still accurately describe the shape of a TDCR with spacer disks when sufficient number disks is used. Similarly, models accounting for partially constrained tendons can be used for TDCR with lumen if the segments are discretized in a sufficient number of subsegments. As a result, there are various options to model a TDCR, that could potentially lead to interesting trade-off between accuracy and computation times.

## 10 Conclusion and Future Research

In this paper, we aim at providing guidelines on choosing a model of TDCR according to the required tradeoff between accuracy and computation time for different design parameters. The existing modelling approaches are described in detail. They can be classified based on the parameterization of the robot backbone and the assumptions on the tendon path. They are then compared on a TDCR design composed of spacer disks, under different loading scenarios and varying design parameters.

A number of interesting results are obtained, including: 1) the superiority of PRB and VC_ref_ models for TDCR with spacer disks in terms of accuracy, 2) the superiority of the CC models in the absence of external forces, for sufficient number of disks 3) the trade-off between accuracy and computation time offered by the CC_sub_ model.

These results inform the user about the suitable choice of model, which will be useful for future research work on TDCRs. In addition, we provide Matlab and C++ implementations of these models to allow the benchmarking of new TDCR models with respect to existing work in the literature.

Implementing and comparing these models raised a number of questions and perspectives that should be addressed in the future. The differences in tendon force expression proposed so far, discussed partly in Section 7.1, needs to be investigated. The concept of subsegment discretization needs to be further analyzed to provide the optimal number of subsegments and the associated geometrical assumptions for a given TDCR. The convergence of the modelling approaches needs also to be investigated to provide insight into selecting an initial guess that will ensure convergence and reduce the computation time. Finally, these models need to be compared to other designs of TDCRs, in particular designs with helical routing where the torsion experienced by the backbone has a significant impact on the robot shape.

## Data Availability Statement

The open source implementations of all the models used in the case studies can be found on https://github.com/SvenLilge/tdcr-modeling.git.

## Author Contributions

PR worked on a major part of paper writing, reviewing the current state-of-the-art and wrote the survey with uniform terminology and notation. She also helped develop the models in MATLAB. QP was responsible for writing parts of the paper, designing and evaluating the case studies, implementing and developing the MATLAB models SL worked on writing parts of the paper and implemented the models in C++. JB-K is the senior author on this paper. She conceptualized, advised, and regularly discussed research questions and results with all authors. She edited the paper. All the authors were concerned with conceptualizing and revising the paper through regular discussions on the theory and implementation details.

## Funding

We acknowledge the support of the Natural Sciences and Engineering Research Council of Canada (NSERC), (RGPIN-2019-04846).

## Conflict of Interest

The authors declare that the research was conducted in the absence of any commercial or financial relationships that could be construed as a potential conflict of interest.
